# In vitro differentiated plasmacytoid dendritic cells as a tool to induce anti-leukemia activity of natural killer cells

**DOI:** 10.1007/s00262-017-2022-y

**Published:** 2017-05-29

**Authors:** Yildian Díaz-Rodríguez, Paulo Cordeiro, Assila Belounis, Sabine Herblot, Michel Duval

**Affiliations:** 1 Unité de recherche en hémato-oncologie Charles-Bruneau, Centre de Recherche du CHU Sainte-Justine, 3175, Côte Sainte-Catherine, Montreal, QC H3T 1C5 Canada; 2 Département de Microbiologie, Infectiologie and Immunologie, Université de Montréal, Montreal, QC Canada; 3 Département de Pédiatrie, Université de Montréal, Montreal, QC Canada

**Keywords:** Cancer immunotherapy, Natural killer cells, Plasmacytoid dendritic cells, In vitro expansion and differentiation of hematopoietic cells, Aryl hydrocarbon receptor antagonist

## Abstract

Acute lymphoblastic leukemia (ALL) is believed to be resistant to NK cell-mediated killing. To overcome this resistance, we developed an innovative approach based on NK cell stimulation with Toll-like receptor (TLR)-activated plasmacytoid dendritic cells (pDC). The translation of this approach into the clinic requires the production of high numbers of human pDC. Herein, we show that in vitro differentiation of cord blood CD34^+^ progenitors in the presence of aryl hydrocarbon receptor antagonists gives rise to clinically relevant numbers of pDC, as about 10^8^ pDC can be produced from a typical cord blood unit. Blocking the aryl hydrocarbon receptor (AHR) pathway significantly increased the yield of pDC. When compared to pDC isolated from peripheral blood, in vitro differentiated pDC (ivD-pDC) exhibited an increased capacity to induce NK cell-mediated killing of ALL. Although ivD-pDC produced lower amounts of IFN-α than peripheral blood pDC upon TLR activation, they produced more IFN-λ2, known to play a critical role in the induction of anti-tumoral NK cell functions. Both TLR-9 and TLR-7 ligands triggered pDC-induced NK cell activation, offering the possibility to use any clinical-grade TLR-7 or TLR-9 ligands in future clinical trials. Finally, adoptive transfer of ivD-pDC cultured in the presence of an AHR antagonist cured humanized mice with minimal ALL disease. Collectively, our results pave the way to clinical-grade production of sufficient numbers of human pDC for innate immunotherapy against ALL and other refractory malignancies.

## Introduction

In the last decade, immunotherapy has been considered a major breakthrough in the field of anti-cancer therapy, since this approach demonstrated its efficacy against chemotherapy refractory cancers. Although many efforts focused on antigen-targeted approaches, harnessing innate immunity to fight cancer cells has also been proposed and natural killer (NK) cells are increasingly used to design anti-cancer immunotherapy [1–3].

NK cells recognize and kill infected or transformed cells without prior sensitization [3]. Their cytotoxicity activity against cancer cells is highly regulated by the balance between activating and inhibitory signals as well as their education in order to distinguish self and untransformed cells from cancer and infected cells. Nonetheless, cancer cells can become resistant to NK cell-mediated lysis by down-regulating ligands for NK cell activating receptors. To circumvent this resistance, NK cell stimulation is required to increase the cytotoxic functions of NK cells [4]. Interleukin (IL)-2 and IL-15 are the most frequently used cytokines to increase NK cell lytic functions, but their use in clinics is associated with high toxicity and side effects that can dampen the efficacy of NK cell-mediated cytotoxicity against cancer. Indeed, concentrations over 20,000 IU/mL commonly used in vitro would be highly toxic in patients [5]. Moreover, lower doses of IL-2 result in regulatory T cell expansion that may reduce immune responses, including NK cell anti-leukemia functions [6–8], and recent data revealed that IL-15 promotes B-acute lymphoblastic leukemia (ALL) cell expansion and their invasion of the central nervous system [9, 10]. NK cell functions can also be stimulated by low numbers of activated dendritic cells [11]. In particular, plasmacytoid dendritic cells (pDC) are an attractive tool to stimulate NK cell functions since this specialized dendritic subset produces high amounts of type I Interferon (IFN) in response to stimulation. Moreover, we recently demonstrated that Toll-like receptor (TLR)-activated pDC induce a unique NK cell phenotype that could not be reproduced by IFN-α alone [12]. This phenotype is characterized by a high expression of TNF-related apoptosis-inducing ligand (TRAIL) on the cell surface.

The outcome for relapsed ALL has not significantly improved over the last 2 decades, despite advances in hematopoietic stem cell transplantation (HSCT). As we and others showed that functional NK cells reappear 1 month after HSCT, these innate immune effectors are an attractive tool to fight residual leukemia early after transplant [13–16]. However, ALL cells are resistant to NK cell-mediated lysis [17, 18]. We recently revealed that co-culture of resting NK cells with TLR-9-activated pDC overcame the resistance of ALL to NK cell killing. Nonetheless, the efficacy of post-transplant administration of a TLR ligand to activate donor-derived pDC may be impaired by the defective pDC reconstitution after HSCT [19]. Adoptive transfer of pDC is therefore a novel therapeutic option to activate NK cells early after HSCT and eradicate residual ALL cells. Thus, we used a preclinical model of HSCT to study the adoptive transfers of pDC and demonstrated that this transfer controlled the development of human ALL in humanized mice [12]. These findings open new therapeutic options for patients with refractory ALL.

The principal drawback for adoptive transfers of activated pDC in the clinic is their low frequency in the peripheral blood, precluding their isolation in sufficient numbers for patient therapy. However, pDC could be expanded and differentiated in vitro from human hematopoietic precursors in the presence of FMS-like tyrosine kinase receptor 3 ligand (FLT3-L) and thrombopoietin (TPO) [19, 20]. In addition, recent reports showed that aryl hydrocarbon receptor (AHR) antagonists favored the differentiation toward the dendritic pathway and increased the numbers of pDC generated from CD34^+^ human progenitors [21]. Nonetheless, the capacity of these in vitro differentiated pDC (ivD-pDC) to induce NK cell cytotoxicity against ALL remains to be determined. We therefore aimed to produce clinically relevant numbers of ivD-pDC from cord blood CD34^+^ cells in the presence of FLT3-L, TPO, and AHR antagonists. We also aimed to characterize ivD-pDC and to evaluate their capacity to induce NK cell cytotoxicity against ALL in vitro and in vivo.

## Materials and methods

### Cell line

The pre-B ALL cell line, REH, was obtained from ATCC (Manassas, VA, USA) and maintained in RPMI-1640 medium (Wisent, Saint-Bruno, QC, Canada) supplemented with 10% heat-inactivated FBS (Wisent) at 37 °C in a 5% CO_2_ atmosphere. This cell line was transduced with a GFP-expressing retrovirus to obtain REH-GFP cells easily traceable by flow cytometry.

### In vitro DC differentiation

Human pDC were expanded and differentiated from purified cord blood CD34^+^ progenitors as previously described [19]. Briefly, cord blood units were obtained from the CHU Sainte-Justine Research Center cord blood bank with the approval from the Institutional Review Board. Mononuclear cells were isolated by gradient centrifugation using Ficoll-Paque Plus (GE Healthcare Bio-Science AB, Uppsala, Sweden), and CD34^+^ cells were positively selected using magnetic beads (Miltenyi Biotec, San Diego, CA, USA). Purified cells were seeded at 0.2 × 10^6^/mL in serum-free expansion medium (StemSpan™ SFEM, StemCell Technologies, Vancouver, BC, Canada), complemented with recombinant human stem cell factor (10 mg/mL), TPO (50 mg/mL), and FLT3-Ligand (100 ng/mL) (all from R&D System, Minneapolis, MN, USA; or Miltenyi Biotec). When required, AHR antagonists were added: CH223191 (1 µM, Sigma, St-Louis, MO, USA) or SR1 (1 µM, Selleckchem, Houston, TX, USA). Culture medium was refreshed every 2–3 days and after 7 days of culture, and culture medium was replaced by StemSpan medium supplemented with human IL-7 (10 ng/mL, Cytheris, Issy-les-Moulineaux, France), TPO, FLT3-L, and CH223191 or SR1. Cultures were maintained for 14 days at 37 °C in a humidified incubator with 5% CO_2_. In vitro differentiated pDC were then purified by flow cytometry after staining with mouse anti-human antibodies (HLA-DR-PE/Cy7 and CD123-APC, BD Biosciences, San Jose, CA, USA) and exclusion of dead cells with Sytox Blue dye. In vitro differentiated mDC were sorted from the same culture using CD1c-PE and HLA-DR-PE/Cy7 markers. Cell sorting was performed on an Aria cell sorter (BD Biosciences). Sorted DC (pDC: HLA-DR^+^/CD123^high^ Sytox^neg^, mDC: HLA-DR^+^/CD1c^+^ Sytox^neg^) were then resuspended in RPMI1640 medium (Wisent) supplemented with 10% of heat-inactivated serum and used for NK cell stimulation experiments.

Monocyte-derived DC (mo-DC) were generated by culturing CD14^+^ peripheral blood monocytes in the presence of GM-CSF (50 ng/mL) and IL-4 (10 ng/mL) (both from R&D systems) for 4 days. Lipopolysaccharide (1 µg/mL) was then added for 2 additional days.

### NK cell and DC isolation from adult peripheral blood

Peripheral blood samples were obtained from healthy volunteers after written informed consent was obtained in accordance with the Declaration of Helsinki and CHU Sainte Justine IRB approval. Peripheral blood mononuclear cells (PBMC) were prepared by density gradient centrifugation using Ficoll-Paque Plus. NK cells, pDC, and mDC were purified by negative selection using magnetic beads (EasySep® enrichment kits, StemCell Technologies). The purity of NK cells and DC was assessed by flow cytometry and was always above 95%.

### NK cell stimulation

Purified NK cells were plated in a 96-well round-bottom plate (2 × 10^6^ cells/mL) and in vitro differentiated- or adult pDC were added in a NK:pDC ratio of 10:1. pDC were stimulated by adding a TLR-9 ligand (CpG-A ODN2216, 10 µg/mL, InvivoGen, San Diego, CA, USA) or a TLR-7 ligand (Imiquimod, 0.8 µg/mL, Sigma), and NK/pDC co-cultures were incubated overnight at 37 °C and 5% CO_2_ atmosphere. Similarly, NK cells were co-cultured with mo-DC, peripheral blood or in vitro generated mDC. mDC and mo-DC were stimulated by adding a TLR-3 ligand (poly-IC, 10 µg/mL, Sigma). Unstimulated NK cells or NK cells cultured with unstimulated pDC were used as negative controls. NK cells stimulated with IFN-α (1000 IU/mL) were used as a positive control for each experiment. Increasing doses of IL-2 (200–20,000 IU/mL; Novartis Pharmaceuticals Canada, Dorval, Quebec, Canada) were also used to stimulate NK cells. IFN-α signaling neutralization assays were performed by incubating NK cells and pDC with neutralizing antibodies (anti-IFN-α/β receptor chain2 and anti-IFN-α) (20 µg/mL, MMHAR-2 and MMHA-2, respectively, PBL Assay Science, Piscataway, NJ, USA) for 30 min prior to the addition of TLR ligands or IFN-α. Blocking antibodies were kept in culture medium overnight.

### Analysis of NK cell activation by flow cytometry

NK cell activation was analyzed after overnight stimulation with IFN-α or activated pDC. Cells were harvested, washed, and then stained with conjugated antibodies: APC-anti-human CD56, PE/Cy5-anti-human CD3 to define NK cell population (CD56^+^CD3^−^), FITC-anti-human CD69, and PE-anti-TRAIL. Activated pDC were labeled with anti-human CD123, anti-human HLA-DR, anti-human CD40, and anti-human CD86 (pDC were defined as HLA-DR^+^/CD123^high^). All conjugated antibodies were purchased from BD Biosciences or Biolegend (San Diego, CA, USA). Samples were analyzed in a LSR Fortessa cytometer (BD Biosciences), and data analysis was performed with FlowJo software version 10 (Tree Star, Ashland, OR, USA).

### NK cell cytotoxic assays

NK cytotoxic assays were performed by using flow cytometry. Briefly, 10^5^ target cells (REH-GFP) and 5 × 10^5^ NK cells were plated in triplicates (effector:target ratio of 5:1) in a 96-well coned plates and incubated for 2 h at 37 °C. Cells were then harvested, dead cells were labeled with propidium iodide (PI), and counting beads were added to each sample. Flow cytometry acquisitions were performed on a LSR Fortessa cytometer (BD Biosciences). The absolute number of live target cells (GFP^+^PI^−^) was calculated, and the percentage of specific lysis was defined as follows: Specific lysis (%) = [(#absolute live target cells–#experimental live target cells)/#absolute live target cells] × 100.

### IFN-α and IFN-γ quantification using enzyme-linked immunosorbent assay (ELISA)

After NK cell stimulation with IFN-α or activated pDC, culture supernatants were collected and stored at −80 °C. IFN-α and IFN-γ quantification was performed by ELISA following the manufacturer’s protocol (PBL InterferonSource, Piscataway, NJ, USA).

### IFN-λ quantification using quantitative PCR

Purified peripheral blood pDC or ivD-pDC were stimulated with ODN CpG 2216 for 24 h. Unstimulated and stimulated cells were harvested, and total RNA contents were prepared using Qiagen RNeasy kit according to manufacturers’ instructions (Qiagen, Hilden, Germany). Quantitative RT-PCR was performed using QuantiTect Probe PCR Kit (Qiagen, Mississauga, ON, Canada). Specific primers and FAM probes for β2 microglobulin, IL-29, and IL-28A were purchased from ThermoFisher Scientific (Waltham, MA, USA). For IL-28B, we used custom primers and FAM probe (Fw: CAAAGATGCCTTAGAAGAGTCG, Rv: TCCAGAACCTTCAGCGTCAG, FAM probe: GCTGAAGGACTGCAAGTGCCG) [22].

### In vivo control of ALL in humanized mice

*Nod*/Scid/*IL*-*2R*γ^−/−^ mice were purchased from the Jackson Laboratory (Bar Harbor, ME) and maintained in pathogen-free conditions. Humanized mice were generated as previously described [12]. Protocols for generating humanized mice were approved by our local Animal Care Committee according to the guidelines of the Canadian Council on Animal Care in Science. To promote human NK cell differentiation, humanized mice received human IL-15/IL-15Rα-Fc complex (ALT803, Altor Biosciences, Miramar, FL, USA [23]) once a week for 7 weeks starting 6 weeks after transplantation. To reproduce human ALL in humanized mice, 5 × 10^3^ REH cells expressing the firefly luciferase gene (Fluc) were injected intravenously 8 weeks after transplantation, followed 48 h later by infusions of unstimulated or TLR-9 activated ivD-pDC (10^5^ cells per mouse). pDC injections were repeated once a week for 5 weeks. A group of humanized mice injected with REH cells was treated by daily injections of human IL-2 (20,000 IU) for 2 weeks. A group of humanized mice injected with REH cells and treated with saline injection was used as control. Leukemia development was monitored by weekly in vivo bioluminescence imaging using a custom apparatus from Labeo Technologies Inc (Montreal, QC, Canada). Briefly, mice were anesthetized and imaged 12 min after intraperitoneal injection of 150 mg/kg D-luciferin (Caliper Life Sciences, Waltham, MA, USA). Each mouse was imaged in anterior–posterior prone position using a constant exposure time (500 ms). Mice were euthanized when overt leukemia signs were observed.

### Statistics

One-way ANOVA tests were used for multiple group comparisons of paired data, and paired *t* tests were used for single data comparisons. The log-rank test was used to compare survival curves. A value of *p* < 0.05 (*) was considered significant with a confidence interval of 99% (GraphPad Software, San Diego, CA, USA).

## Results

### AHR antagonists increase in vitro differentiation of functional plasmacytoid dendritic cells from CD34^+^ progenitors

We and others showed that the culture of human CD34^+^ progenitors, in the presence of FLT3-L and TPO, gave rise to fully differentiated pDC [12, 20, 24]. Additionally, the presence of AHR antagonists favored dendritic cell differentiation [21, 25]. Since the induction of differentiation programs may also interfere with the cellular functions of in vitro generated cells, we aimed to assess the functional properties of human ivD-pDC obtained in the absence or in the presence of AHR antagonists (CH223191 and StemRegenin-1—SR1).

**Fig. 1:**
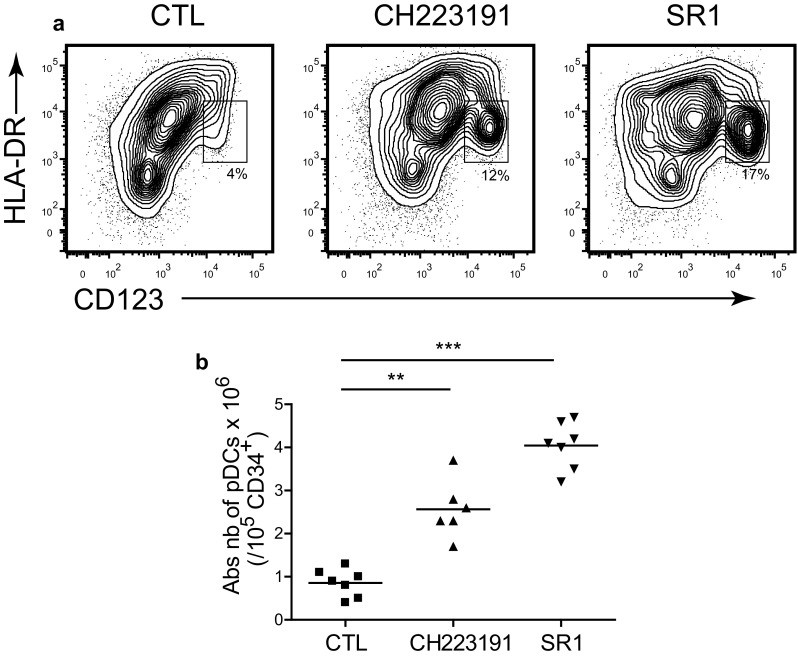
AHR antagonists increase the yield of in vitro differentiated pDC. Cord blood CD34^+^ progenitors were cultured for 2 weeks in the absence (CTL—control) or in the presence of AHR antagonists (CH223191 or StemRegenin-1, SR1). Absolute numbers of differentiated pDC were determined by using immunostaining and flow cytometry analysis. **a** Differentiated pDC were identified as HLA-DR^+^CD123^hi^ by flow cytometry. Representative contour plots are presented with percentage of pDC for each culture condition. **b** Absolute *numbers* of pDC obtained from 10^5^ CD34^+^ cells are presented with median (*n* = 7 independent experiments). Statistical analyses were performed by using one-way analysis of variance. **p < 0.01, ***p < 0.001

Human CD34^+^ progenitors were purified from cord blood samples and cultured for 2 weeks with or without AHR antagonists [12]. The presence of AHR antagonists increased both percentages and absolute numbers of ivD-pDC, and SR1 was superior to CH223191 to induce pDC differentiation (Fig. 1). We were able to obtain an average number of 4 × 10^6^ ivD-pDC from 10^5^ CD34^+^ cord blood cells.

**Fig. 2:**
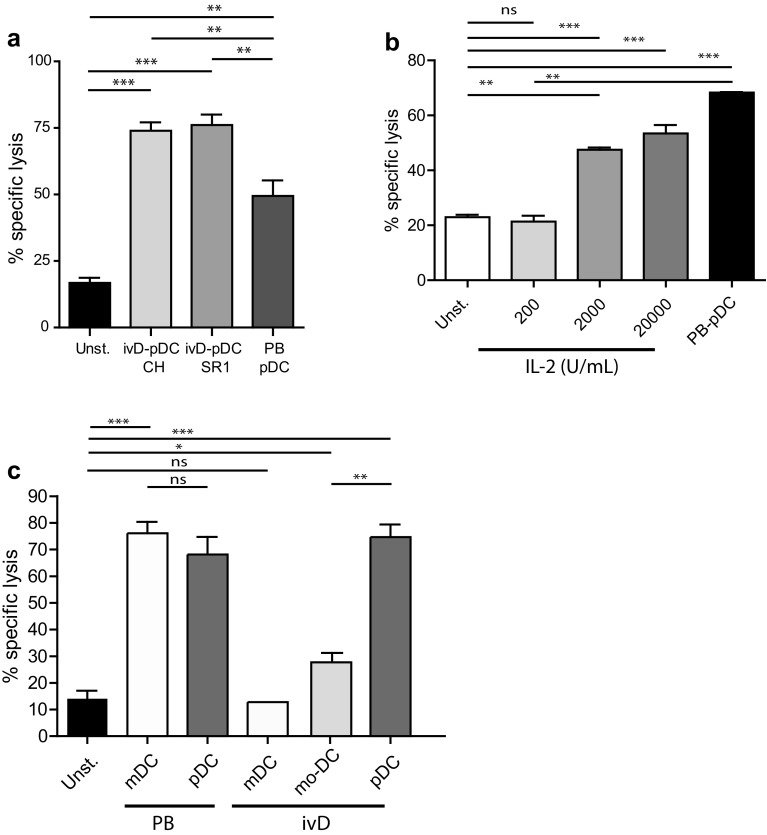
TLR-9-activated ivD-pDC induce NK cell cytolytic activity against ALL that cannot be reproduced by IL-2 or ivD-mDC stimulation of NK cells. Peripheral blood NK cells were stimulated by 18-h co-culture with ivD-pDC or PB-pDC in the presence of TLR-9 ligand (CpG ODN 2216, 10 µg/mL) or unstimulated (unst.). **a** Cytotoxic assays were performed against REH pre-B ALL cell line at a E:T ratio of 5:1 using flow cytometry count of viable cells. Averages of specific lysis are presented with standard deviation (SD) (*n* = 5 independent experiments). **b** Peripheral blood NK cells were unstimulated (unst.) or stimulated by 18-h co-culture with PB-pDC in the presence of CpG ODN 2216 (10 µg/mL) or increasing amount of IL-2. Cytotoxic assays were performed against REH pre-B ALL cell line at a E:T ratio of 5:1. Averages of specific lysis are presented with standard deviation (SD) (*n* = 3 independent experiments). **c** Peripheral blood NK cells were unstimulated (unst) or stimulated by 18-h co-culture with PB-mDC or PB-pDC or in vitro differentiated mDC, mo-DC or pDC. The TLR-9 ligand CpG ODN 2216 (10 µg/mL) was added in the culture containing pDC, and the TLR-3 ligand Poly(I:C) (1 µg/mL) was added in the culture containing mDC. Cytotoxic assays were performed against REH cells at a E:T ratio of 5:1. Averages of specific lysis are presented with standard deviation (SD) (*n* = 2–3 independent experiments). Statistical analyses were performed by using one-way analysis of variance. *p < 0.05, **p < 0.01, ***p < 0.001

We next assessed the competence of ivD-pDC to induce NK cell-mediated killing of leukemia. IvD-pDC were sorted by flow cytometry according to HLA-DR and CD123 surface expression. Sorted cells were co-cultured with purified peripheral blood NK cells in the presence of a TLR-9 ligand (A-CpG ODN) at a pDC:NK cell ratio of 1:10. Cytotoxic assays against a pre-B ALL cell line using unstimulated NK cells or pDC-activated NK cells revealed that NK cell cytotoxicity against ALL was not affected by the presence of AHR antagonists in pDC culture. The killing of ALL cells was even significantly higher with ivD-pDC than with pDC isolated from peripheral blood (PB-pDC), reaching an average of 75% ALL cell killing at a NK:ALL ratio of 5:1 (Fig. 2a).

At concentrations attainable in humans (200 IU/mL), stimulation of NK cells with IL-2 was not able to induce ALL lysis (Fig. 2b). High doses of IL-2 (20,000 IU/mL) induced 55% ALL cell killing at a NK:ALL ratio of 5:1, but these concentrations would be highly toxic in patients. We also compared ALL lysis induced by pDC-stimulated NK cells and by mDC-stimulated NK cells. mDC were either purified from peripheral blood (PB-mDC), in vitro generated from cord blood CD34^+^ cells (ivD-mDC), or monocytes (mo-DC) and stimulated with the TLR-3 ligand Poly(I:C). NK cell-mediated cytotoxicity against REH cells was similar for NK cells stimulated with activated PB-mDC and PB-pDC (Fig. 2c). Nonetheless, in vitro generated mDC (either mo-DC or ivD-mDC) were unable to induce NK cell-mediated ALL cell lysis (Fig. 2c).

**Fig. 3:**
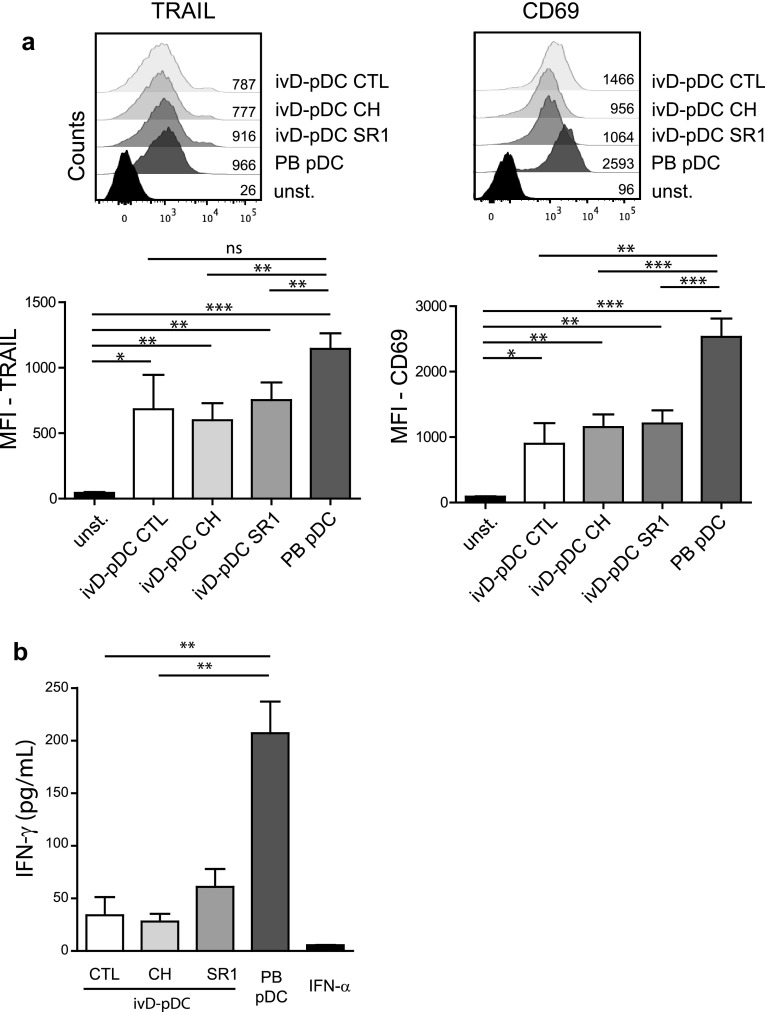
Phenotypic changes of pDC-stimulated NK cells. **a** Phenotypic analysis of activated NK cells was performed by immunostaining (TRAIL and CD69) and flow cytometry analysis (gated on CD56^+^CD3^−^ cells). Representative histograms are presented with mean of fluorescence (MFI) for TRAIL (*left panel*) and CD69 (*right panel*). *Bar graphs* represent the average MFI for TRAIL and CD69 with SD (*n* = 3–7 experiments). **b** IFN-γ production by unstimulated NK cells (CTL—control) or pDC-stimulated NK cells was measured by ELISA in culture supernatants. Averages of 6 independent experiments are presented with SD. Statistical analyses were performed by using one-way analysis of variance. *p < 0.05, **p < 0.01, ***p < 0.001

Although the induction of NK cell cytotoxicity was stronger with ivD-pDC than with PB-pDC, there were some phenotypic differences between NK cells stimulated by ivD-pDC and by PB-pDC. TLR-9-activated ivD-pDC induced the up-regulation of CD69 and TRAIL on NK cells, although this up-regulation was lower than that observed after NK cell stimulation with TLR-9-activated PB-pDC (Fig. 3a). The production of IFN-γ by NK cells stimulated with ivD-pDC was also four- to fivefold lower than by NK cells stimulated with PB-pDC (Fig. 3b). TRAIL and CD69 expression and IFN-γ production by NK cells were the same for all ivD-pDC, whether they were obtained in the presence of AHR or not. Taken together, these results indicate that ivD-pDC are more potent than PB-pDC to induce NK cell lytic activity against ALL, despite a lower expression of TRAIL and CD69 on NK cells and weaker IFN-γ production.

### IvD-pDC produce less IFN-α, but more IFN-λ2 than PB-pDC

**Fig. 4:**
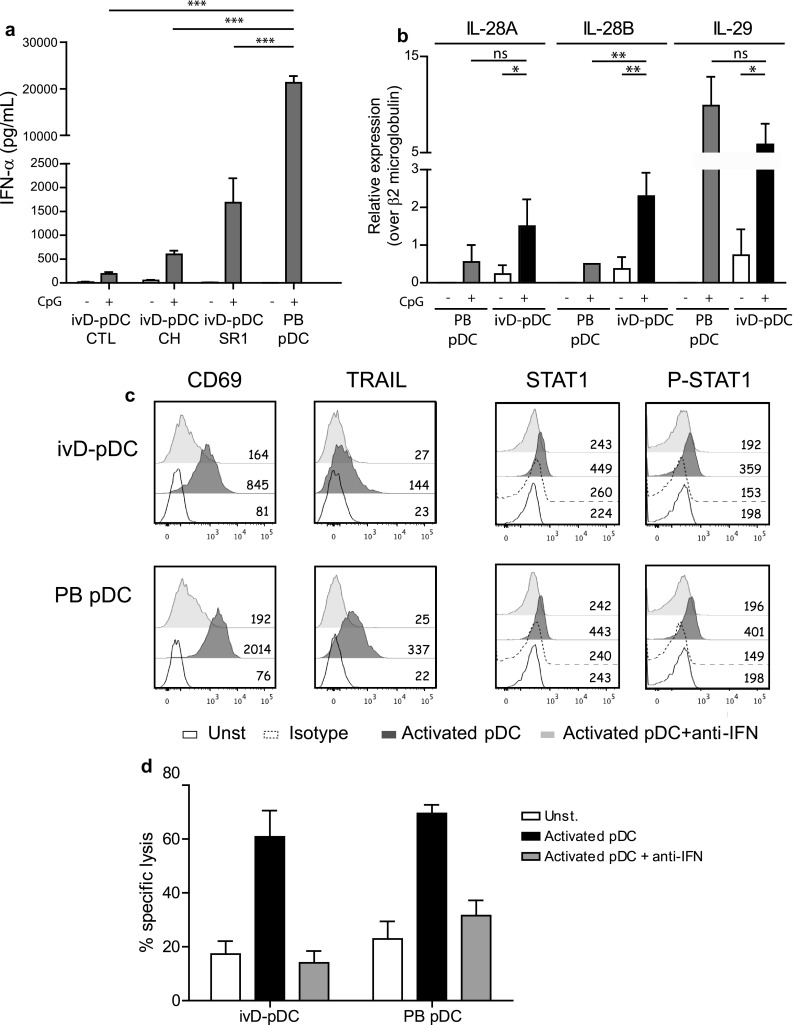
IFN-α signaling is required for NK cell stimulation by ivD-pDC although ivD-pDC produce less IFN-α as compared with PB-pDC. **a** The production of IFN-α was assessed by ELISA in culture supernatants following stimulation of purified pDC with a TLR-9 ligand (CpG ODN 2216, 10 µg/mL). **b** The production of type III IFN (IL-28A, IL-28B, and IL-29) was assessed by Q-PCR before and after TLR stimulation of purified pDC. **c** Type I IFN signaling blockade was performed by using a combination of anti-IFN-α and anti-IFN receptor antibodies in NK/pDC co-cultures. Intracellular staining of STAT1 and phosphorylated-STAT1 confirms the blockade of type I IFN signaling in both ivD-pDC and PB-pDC. This blockade abolishes the up-regulation of TRAIL and CD69 on NK cells. **d** Cytotoxic assays were performed against REH cell line at a ratio E:T 5:1 using unstimulated NK cells, NK cells stimulated with activated ivD-pDC (cultured in the presence of SR1) or PB-pDC, in the presence or the absence of type I IFN blocking antibodies. The mean of specific lysis is presented with SD (*n* = 3 independent experiments). Statistical analyses were performed by using one-way analysis of variance. *p < 0.05, **p < 0.01, ***p < 0.001

We next assessed the production of IFN-α in response to TLR-9 stimulation of ivD-pDC. We observed that although ivD-pDC produced appreciable amounts of IFN-α (up to 2000 pg/mL for 40,000 pDC), these amounts were about tenfold lower than IFN-α produced by PB-pDC (Fig. 4a). Of note, ivD-pDC obtained in the presence of AHR antagonists produced higher amounts of IFN-α than ivD-pDC obtained in the absence of inhibitors, and SR1 was superior to CH223191. We next explored the expression of *IFN*-*λ* (*IL*-*28A*, *IL*-*28B*, and *IL*-*29*) RNA by activated ivD-pDC because it has been recently reported that these type III IFN, particularly IL-28B/IFN-*λ*2, play a major role in NK cell anti-tumoral functions [26–28]. TLR-9 stimulation increased the expression of type III *IFN* RNA in both ivD-pDC and PB-PDC. When compared with activated PB-pDC, activated ivD-pDC expressed as much IL-28A and IL-29, but more *IL*-*28B*/*IFN*-*λ2* RNA (Fig. 4b). These results indicate that the cytotoxic activity of pDC-activated NK cells against ALL cells does not correlate with the amount of IFN-α produced by activated pDC in NK/pDC co-cultures and suggest a role for type III IFN in pDC-induced NK cell activation.

### IFN-α signaling is required for NK cell stimulation by ivD-pDC

Since ivD-pDC produce lower amount of IFN-α than peripheral blood pDC in response to TLR stimulation, we next tested whether type I IFN signaling was required for NK cell response. We used a combination of blocking antibodies against type I IFN receptor and IFN-α to block IFN-α signaling pathway during NK cell stimulation with activated pDC. We verified the efficacy of the blocking antibodies using STAT1 and phosphorylated STAT1 staining and showed that STAT1 signaling was abrogated by our combination of blocking antibodies (Fig. 4c). We observed that IFN-α signaling was required for both TRAIL and CD69 up-regulation on activated NK cells (Fig. 4c) and to induce the cytolytic activity of activated NK cells against ALL (Fig. 4d). Therefore, IFN-α production by activated pDC is required for NK cell stimulation and the lower amount of IFN-α produced by ivD-pDC is sufficient to obtain maximal NK cell lytic activity.

### TLR-7 and TLR-9 ligands are equally efficient to stimulate ivD-pDC and to induce NK cell cytotoxicity against ALL

**Fig. 5:**
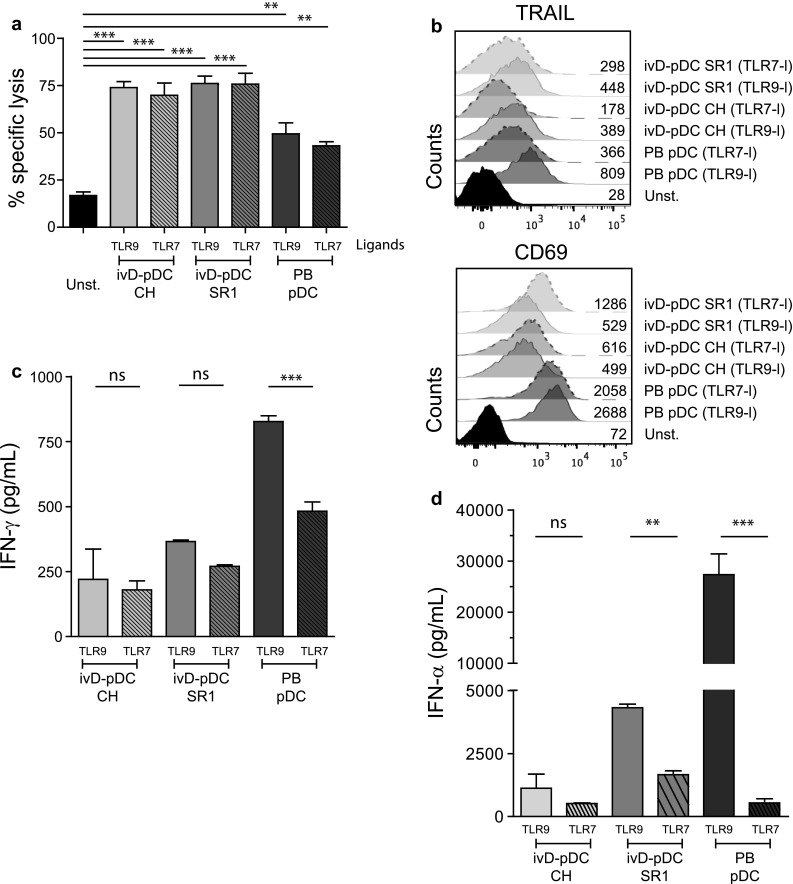
TLR-7 and TLR-9 ligands are equally efficient to stimulate ivD-pDC and to induce NK cell lysis of ALL. We compared the efficacy of TLR-9 (CpG ODN 2216) and TLR-7 (Imiquimod) ligands for the induction of NK cell lytic functions. **a** Cytotoxic assays against REH cells were performed as above with NK cells either unstimulated (unst.) or co-cultured with ivD-pDC or PB-pDC stimulated with TLR-9 or TLR-7 ligand. Mean of specific lysis with SD is presented (*n* = 3 independent experiments). **b** NK cell activation status was assessed by immunostaining and flow cytometry analysis (TRAIL and CD69 markers). **c** IFN-γ production by NK cells was assessed by ELISA in the supernatants of NK/pDC co-cultures in which pDC were stimulated with TLR-9 or TLR-7 ligand. The means of 3 independent experiments are presented with SD. **d** IFN-α production by activated pDC was assessed by ELISA in the culture supernatant of pDC stimulated with TLR-9 or TLR-7 ligand. The mean of 3 independent experiments is presented with SD. Statistical analyses were performed by using one-way analysis of variance. *p < 0.05, **p < 0.01, ***p < 0.001

We then compared pDC stimulation via TLR-7 and TLR-9 pathways for the induction of NK cell activation and NK cell-mediated lysis of ALL cells. Cytotoxic assays against REH cell line did not show any differences in the cytolytic activity of NK cells stimulated with TLR-7- or TLR-9-activated ivD-pDC (Fig. 5a). TRAIL and CD69 expression was induced on NK cells co-cultured with TLR-7- and TLR-9-activated ivD-pDC (Fig. 5b). The production of IFN-γ by activated NK cells was similar when NK cells were co-cultured with TLR-7- or TLR-9-activated ivD-pDC, although PB-pDC activation through TLR-9 induced more IFN-γ production by NK cells than activation through TLR-7 (Fig. 5c). Finally, the production of IFN-α by TLR-7-activated pDC was lower than by TLR-9-activated pDC (Fig. 5d). Taken together these results indicate that, despite a lower production of IFN-α by TLR-7-activated ivD-pDC, TLR-7 and TLR-9 ligands induce similar ivD-pDC-mediated NK cell lytic activity against ALL cells.

### Adoptive transfers of activated ivD-pDC cure minimal ALL disease in humanized mice

**Fig. 6:**
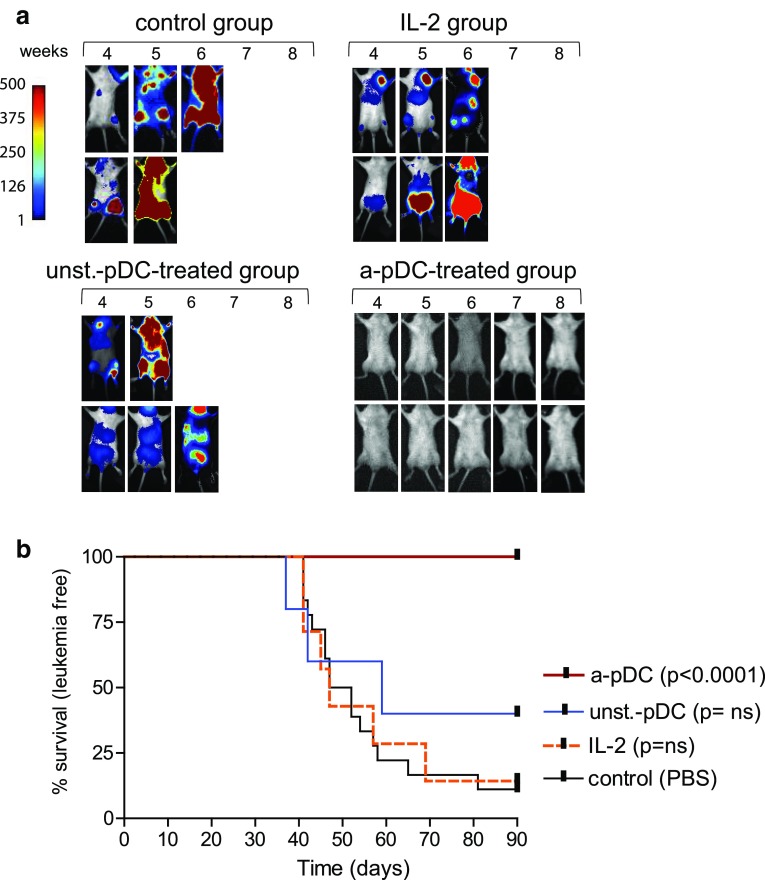
Activated ivD-pDC infusions cure human ALL in humanized mice. Irradiated newborn NSG mice were transplanted with 10^4^ human cord blood-derived CD34^+^ cells. 10^5^ REH ALL cells were intravenously injected in humanized mice 8 weeks after transplantation. Forty-eight hours after leukemia injection, mice were injected with activated pDC (a-pDC; *n* = 12) or unstimulated pDC (unst-pDC, *n* = 5) (once a week, for 5 weeks), 20,000 IU of IL-2 (daily injections; *n* = 7) or saline solution for control mice (control; *n* = 18). **a** In vivo bioluminescence imaging of ALL-bearing mice was performed weekly. *Images* of representative mice are shown; units in *rainbow color scales* are proportional to the numbers of photons per second. **b** Survival *curves* of ALL-bearing humanized mice treated with unstimulated or TLR9-activated ivD-pDC, IL-2 or saline solution injections. Mice were euthanized after overt leukemia onset. Flow cytometry analysis of bone marrow samples confirmed complete leukemia involvement. Log-rank test was used to compare survival

We recently reported that adoptive transfers of activated pDC control human ALL in humanized mice [12]. In these experiments, activated pDC were obtained from in vitro differentiation of CD34^+^ progenitors in the absence of AHR antagonists. We show here that adoptive transfers of TLR-activated ivD-pDC obtained in the presence of the AHR antagonist SR1 cured ALL bearing mice (Fig. 6). To reproduce a minimal ALL disease, we injected 5 × 10^3^ ALL cells in humanized NSG mice. The time to overt leukemia in untreated mice was longer when compared to our previous published data in which 10^4^ ALL cells were injected (52-day median survival versus 35 days), but 85% of control mice died from overt leukemia within 40–90 days post-ALL injection. None of the mice treated with weekly injections of TLR-activated ivD-pDC (10^5^ cells per week −5 weeks) developed leukemia as assessed by in vivo bioluminescence imaging and necropsy at the time of euthanasia (Fig. 6a, b). As controls, we treated a group of humanized mice injected with REH cells with daily injections of recombinant human IL-2 (20,000 IU) and another group of mice with unstimulated ivD-pDC. Mice within these two groups develop leukemia with the same kinetics as untreated control mice (Fig. 6). Therefore, these results demonstrate that adoptive transfer of ivD-pDC obtained in the presence of AHR antagonist is a unique therapeutic tool to cure minimal residual ALL disease in vivo.

## Discussion

Our data show that NK cell stimulation with TLR-activated ivD-pDC induces anti-leukemia activity against resistant ALL cells both in vitro and in vivo. pDC obtained by in vitro differentiation of CD34^+^ progenitors in the presence of AHR antagonists are even more efficient than PB-pDC to stimulate NK cell lytic activity despite lower production of IFN-α and lower expression of NK cell activation markers. We further show that, in the presence of AHR antagonists, clinically relevant numbers of ivD-pDC are obtained from cord blood CD34^+^ progenitor cultures. Both TLR-7 and TLR-9 ligands are equally efficient to stimulate ivD-pDC and induce NK cell anti-leukemia activity. Finally, adoptive transfers of ivD-pDC obtained in the presence of AHR antagonist cured ALL in humanized mice.

We took advantage of the combination of FLT3-L, TPO, and AHR antagonist to produce clinically relevant numbers of ivD-pDC from cord blood CD34^+^ cells. FLT3-L plays a non-redundant role in pDC differentiation, as demonstrated by the lack of pDC in *flt*3-knockout mice and the increase in pDC numbers in humans following injections of recombinant FLT3-L [29–33]. Accordingly, in vitro culture of cord blood-derived hematopoietic progenitors in the presence of FTL3-L gives rise to differentiated DC populations among which pDC represent 3–5% of total cells [34]. Moreover, the addition of TPO increased the yield of human ivD-pDC [20]. Recent studies revealed that AHR inhibitors induce not only human stem cell expansion, but also favor dendritic differentiation and functions [21, 35]. Thus, we associated FLT3-L and AHR to stem cell factor, TPO and IL-7, to produce ivD-pDC. We obtained 40 times more pDC than the initial number of CD34^+^ cells. From the average of 2.5 × 10^6^ CD34^+^ cells contained in a typical cord blood unit, we thus expect to produce 10^8^ ivD-pDC. This number is sufficient for clinical use in one patient, as we were able to cure ALL in mice with 5 injections of 10^5^ pDC. According to the most accurate method of dosage conversion from mice to humans based on body surface area (BSA) [36, 37], 5 injections of 10^5^ pDC correspond to 7.5 × 10^7^ pDC/m^2^, while a 30-kg child BSA is 1 m^2^ and a medium-size adult BSA is 1.7 m^2^, suggesting that about 10^8^ ivD-pDC will be sufficient for human therapy.

NK cell stimulation with high doses of IL-2 or PB-mDC induced NK cell-mediated lysis of ALL, but none of these approaches has the potential for translation into the clinics. Indeed, the concentration of IL-2 required to induce NK cell lytic activity against ALL cells in vitro would be highly toxic in humans, and lower concentrations that can be reached in patients did not induced NK cell lysis of ALL [5]. Accordingly, IL-2 administered to humanized mice at therapeutic doses was unable to control ALL development. Similarly, PB-pDC and PB-mDC are equally efficient to induce NK cell lytic activity, but their low amounts in the peripheral blood of healthy volunteers preclude their use to stimulate NK cell anti-leukemia function in patients. IvD-pDC, ivD-mDC, and mo-DC can be obtained in sufficient numbers for clinical use, but among them only ivD-pDC were able to induce NK cell-mediated killing of ALL.

The equal efficiency of TLR-9 and TLR-7 to activate ivD-pDC is an asset for future clinical use. Indeed, production of clinical-grade activated pDC will require the use of a clinical-grade TLR ligand. Both TLR-7 and TLR-9 ligands are currently in clinical development (NCT02188810 [38, 39]), but the final availability of these compounds for clinical use will depend on the results of ongoing clinical trials. Equal efficacy of different TLR ligands indicates that the possibility to activate ivD-pDC in future clinical trials will not rely on the availability of a single compound.

Activated ivD-pDC produce less IFN-α than activated PB-pDC, and even less when they are activated through the TLR-7 pathway. However, this lower production did not impact the cytolytic activity of NK cells against ALL cells. Indeed, ALL killing was higher when NK cells were activated with ivD-pDCs, reaching an average killing of 75% at a NK:ALL cell ratio of 5:1. This is in line with the results of our previous work on the anti-leukemic potential of PB-pDC [12]. We showed that NK cell activation by pDC was dependent on the IFN-α pathway, but not reproduced by IFN-α alone, suggesting that the paracrine–autocrine activation loop of IFN-α and other cytokines play a role in NK cell activation [40]. Recent results showed the importance of the IFN-*λ* pathway, and particularly of IFN-*λ*2 (IL28-B), in NK cell function and anti-cancer activity [26–28]. Thus, we explored the expression of type III/*IFN*-*λ* (*IL*-*28A*, *IL*-*28B*, and *IL*-*29*) RNA following TLR activation of pDC. IL28-A and IL-29 expression was not significantly different between PB-pDC and ivD-pDC, but IL28-B/IFN-*λ*2 expression was higher in ivD-pDC. As we showed that NK cell activation by pDC was independent of cell contact [12], type III IFN and particularly IL28-B/IFN-*λ*2 are good candidates as the soluble mediators of NK cell activation. Experiments are underway to further delineate their role as well as the role of other cytokines.

Collectively, our results pave the way to clinical-grade production of sufficient numbers of human pDC for therapeutic use. Our serum-free culture conditions will be easily compatible with GMP standards, and most of the clinical-grade TLR-7 or TLR-9 ligands are expected to be as efficient to induce ivD-pDC activation. This novel immunotherapeutic approach based on early post-transplant NK cell stimulation by adoptive transfers of ivD-pDC eradicates the residual leukemic cells and prevents the relapse of leukemia in a preclinical model. It may even be used in other types of cancer, as we have recently showed its efficacy against neuroblastoma, a leading cause of death from cancer in children.

## Abbreviations

AHR: Aryl hydrocarbon receptor
ALL: Acute lymphoblastic leukemia
ATCC: American Type Culture Collection
BSA: Body surface area
CD: Cluster of differentiation
DC: Dendritic cells
ELISA: Enzyme-linked immunosorbent assay
FBS: Fetal bovine serum
FITC: Fluorescein isothiocyanate
Fluc: Firefly luciferase
FLT3-L: FMS-like tyrosine kinase receptor 3 ligand
GFP: Green fluorescent protein
GM-CSF: Granulocyte macrophage-colony stimulating factor
HSCT: Hematopoietic stem cell transplantation
IFN: Interferon
IL: Interleukin
IU: International unit
ivD-: In vitro differentiated-
mDC: Myeloid dendritic cells
mo-DC: Monocyte-derived dendritic cells
NK: Natural killer
NSG: *Nod*/*Scid*/γc^−/−^
ODN: Oligonucleotide
PB: Peripheral blood
PBMC: Peripheral blood mononuclear cells
pDC: Plasmacytoid dendritic cells
PE: Phycoerythrin
PI: Propidium iodide
Q-PCR: Quantitative polymerase chain reaction
RPMI: Roswell Park Memorial Institute medium
SR1: StemRegenin 1
TLR: Toll-like receptor
TNF: Tumor necrosis factor
TPO: Thrombopoietin
TRAIL: TNF-related apoptosis-inducing ligand
